# Identification of QTL underlying the leaf length and area of different leaves in barley

**DOI:** 10.1038/s41598-019-40703-6

**Published:** 2019-03-14

**Authors:** Binbin Du, Lipan Liu, Qifei Wang, Genlou Sun, Xifeng Ren, Chengdao Li, Dongfa Sun

**Affiliations:** 1 College of Plant Science and Technology, Huazhong Agricultural University, Wuhan, 430070 China; 2 Biology Department, Saint Mary’s University, 923 Robie Street, Halifax, NS B3H 3C3 Canada; 3 Department of Agriculture & Food/Agricultural Research Western Australia, 3 Baron-Hay Court, South Perth, WA 6155 Australia; 4 Hubei Collaborative Innovation Center for Grain Industry, Yangtze University, Jingzhou, 434025 Hubei China

## Abstract

Leaf is the main organ of photosynthesis, which significantly impacts crop yield. A high-density linkage map containing 1894 single nucleotide polymorphism (SNP) and 68 simple sequence repeats (SSR) markers was used to identify quantitative trait locus (QTL) for flag leaf length (FLL), second leaf length (SLL), third leaf length (TLL), fourth leaf length (FOLL), flag leaf area (FLA), second leaf area (SLA), third leaf area (TLA) and fourth leaf area (FOLA). In total, 57 QTLs underlying the top four leaf length and area traits were identified and mapped on chromosome 2H, 3H, 4H and 7H. Individual QTL accounted for 5.17% to 37.11% of the phenotypic variation in 2015 and 2016. A major stable QTL *qFLL*2*-*2 close to the marker 2HL_25536047 was identified on the long arm of chromosome 2H. The most important QTL clustered region at M_256210_824 - 2HL_23335246 on chromosome 2H was associated with FLL, SLL, FLA and SLA and explained high phenotypic variation. These findings provide genetic basis for improving the leaf morphology of barley. In addition, our results suggested that the top four leaves were significantly positively correlated with plant height and some yield-related traits.

## Introduction

Barley (*Hordeum vulgare* L.), as one of the earliest domesticated and most important cereal crops for humanity, is widely used for food, feed and malting. Leaf is the main organ of photosynthesis and plays a crucial role in determining yield of crops^1^. In cereals, the upmost three leaves, particularly the flag leaf, assimilate the most light energy and are main source of generation for carbohydrate production^2–4^. The top two leaves produce over 80% photosynthesis of the whole plant during grain filling^4^. Moreover, leaf size and shape are regarded as important traits determining photosynthesis capability and grain yield^5–7^. Therefore, it is necessary to disclose the genetic mechanism of leaf characteristics.

QTL underlying leaf morphological traits has been identified in some crops^8–11^, such as QTL controlling flag leaf length in rice^8^, QTL for flag leaf size and morphology in wheat^10^.

To date, the morphological traits of flag leaf have been widely studied in barley and were determined by polygenes with vulnerability to influence from environment^12–18^. Digel *et al*.^19^ identified PHOTOPERIOD-H1 (Ppd-H1) as a candidate gene controlling leaf size in barley. Xue *et al*.^17^ reported QTLs for FLL and FLW on chromosome 5H and 7H using a comprehensive DArT and SSR genetic map. Liu *et al*.^18^ identified two pleiotropic genomic regions on chromosome 2H and 7H controlling FLL, FLW, FLA and some physiological traits. The limitation of previous studies was the limited number of markers used. High-density map can improve the accuracy of QTL and precise location of important traits^20^.

Our group has constructed a high density of linkage map containing 1962 SNP and SSR markers using a barley DH population^21^, which has previously been used for identifying QTLs underlying yield-related traits^22^, physiological traits of flag leaf including net photosynthesis rate^18^ and mapping a new dwarf gene *btwd*1^23^. The objectives of this study were to use this high density of linkage map to identify QTL associated with the topmost four leaf length and area traits; and to reveal the relationships among the topmost four leaves, plant height and yield-related traits. The detected QTLs and their closely linked markers can be used for marker-assisted selection (MAS) in barley breeding.

## Methods

### Experimental materials and field trial

The 122 doubled haploid (DH) lines from a cross between dwarf-barley cultivar Huaai11 and feed-barley cultivar Huadamai6 were obtained^21^. Huaai11 is a six-rowed, dwarf-barley cultivar derived from barley landrace, Daofu Baiqingke. Huadamai6 is a two-rowed, feed-barley cultivar. In addition to the differences in plant height between two parents, they also showed distinct difference in leaf length and area traits. The DH lines and parents were sown in a plot with line length of 1.5 m and line interval of 0.2 m at the experimental farm of Huazhong Agricultural University, Wuhan, China in 2015 and 2016. The field trial followed a completely randomized block design, with 3 replications each year. Each DH and parental line was grown in two rows with eight seedlings in each row.

### Trait measurement

At the stages of pre-filling and with fully unfolded flag leaves on the main stem, 6 plants in the middle of rows from each replication were randomly chosen to measure 8 morphological traits including flag leaf length (FLL, cm), second leaf length (SLL, cm), third leaf length (TLL, cm) and fourth leaf length (FOLL, cm), as well as flag leaf area (FLA, cm^2^), second leaf area (SLA, cm^2^), third leaf area (TLA, cm^2^) and fourth leaf area (FOLA, cm^2^). The leaf length was examined from leaf base to tip. Leaf area was calculated using width × length × 0.75^24^, in which the length and width were measured on the widest and longest part of the blade, respectively. The measurement of plant height (PH) and yield-related traits of all lines including main spike length (MSL), grain number per plant (GP), spikelet number per plant (SLP), grain weight per spike (GWS), grain weight per plant (GWP) and thousand grain weight (TGW) was described by Wang *et al*.^22^.

### Phenotypic data analysis

The mean values of each year were used for statistics, correlation analysis and QTL mapping. Descriptive statistics of all phenotypic data were performed using IBM SPSS Statistics 22 software. Correlation analysis between traits and their significance levels were calculated using the Pearson’s correlation coefficients analysis method and two-tailed T test. The broad-sense heritability value was calculated using *h*_*B*_^2^ = *σ*_*g*_^2^*/(σ*_*g*_^*2*^ + *σ*_*ge*_^2^*/n* + *σ*_*e*_^2^*/rn)*, where the genotype variance σ_g_^2^ = (MS_g_ − MS_ge_)/rn, genotype and environment interaction variance σ_ge_^2^ = (MS_ge_ − MS_error_)/r, error variance σ_e_^2^ = MS_error_. MS_g_, MS_ge_ and MS_error_ represent genotype mean square, genotype and environment interaction mean square and error mean square, respectively, *r* and *n* represent the number of replicates of each genotype and environments, respectively. The variance of the components was estimated using a general linear model (GLM).

### QTL analysis

The genetic map containing 1894 SNP and 68 SSR markers^21–23^ was used to identify QTL using WinQTLcart v2.5 with composite interval mapping^25^. The window size and walking speed were set to 10 cM, and 1 cM, respectively. After performing 1000 permutations, a LOD (Logarithm of the odds) threshold was obtained^26^. The QTL with LOD score greater than LOD threshold was considered to be an important QTL using the significance level (P = 0.05) as the LOD threshold. QTL was considered as minor or major, depending on the phenotypic variance in a primary genetic analysis, if the phenotypic variance was more than 15% which was considered as major QTL^27^. QTL nomenclature followed the principle of McCouch^28^. The QTL location on the map was drawn using MapChart ver. 2.2 software^29^.

## Results

### Phenotypic data evaluation

Phenotypes of the parents and the distribution among DH lines for FLL, SLL, TLL, FOLL, FLA, SLA, TLA, and FOLA in two consecutive years (year 2015 and 2016) were shown in Table 1. The values of all traits in Huadamai 6 were higher than those in Huaai 11, and the two parents showed significant differences in all traits (p < 0.01). The frequency distributions of those traits in two years were consecutive (Supplementary Fig. S1), and the values of skewness and kurtosis for all traits were among −1 and 1, indicating all traits controlled by multiple genes (Table 1). ANOVA showed that genotypic effects of 122 DH lines and parents were significant difference for all traits (p < 0.01). Year effects were significant (p < 0.05) for all traits except SLL, FOLL and SLA. Genotype × year interactions were also significant (p < 0.05) for all traits except SLA. (Table 2). The variable coefficients varied from 13.57% to 33.94% in 2015, and 11.12% to 30.89% in 2016. The broad-sense heritability (*h*_*B*_^2^) of all traits ranged from 88.54% to 91.11%, indicating that genetic factors played an important role in determining these traits.

**Table 1:** The statistics of the 122 lines from DH population and parents for top four leaves length and area traits.

Traits	Year	Huadamai6	Huaai11	ST	DH lines					
		Mean	Mean		Mean	SD	Skew	Kurtosis	CV/%	*h*^*2*^/%
FLL (cm)	2015	15.93 ± 0.55	11.57 ± 0.47	0.000^**^	12.02 ± 0.21	2.35	0.20	−0.59	19.55	88.39
	2016	17.07 ± 0.53	13.18 ± 0.40	0.000^**^	14.32 ± 0.26	2.87	0.51	−0.08	20.04	
SLL (cm)	2015	21.76 ± 0.32	15.15 ± 0.40	0.000^**^	17.68 ± 0.22	2.40	0.30	−0.34	13.57	89.83
	2016	24.16 ± 0.70	16.74 ± 0.32	0.000^**^	19.05 ± 0.24	2.62	0.45	−0.01	13.75	
TLL (cm)	2015	20.95 ± 0.27	15.01 ± 0.30	0.000^**^	17.67 ± 0.22	2.45	0.56	0.43	13.87	89.07
	2016	21.88 ± 0.80	17.36 ± 0.28	0.000^**^	18.88 ± 0.19	2.10	−0.03	−0.60	11.12	
FOLL (cm)	2015	20.32 ± 0.39	14.62 ± 0.32	0.000^**^	16.53 ± 0.23	2.53	0.27	−0.33	15.31	90.24
	2016	22.23 ± 0.35	15.67 ± 0.33	0.000^**^	18.43 ± 0.20	2.23	0.22	−0.66	12.10	
FLA (cm^2^)	2015	18.84 ± 0.77	15.02 ± 0.60	0.000^**^	12.64 ± 0.39	4.29	0.52	−0.40	33.94	88.54
	2016	23.07 ± 0.93	17.72 ± 0.56	0.002^**^	17.45 ± 0.49	5.39	0.73	0.57	30.89	
SLA (cm^2^)	2015	32.61 ± 0.76	21.28 ± 1.06	0.000^**^	24.62 ± 0.46	5.10	0.50	0.29	20.71	90.96
	2016	33.84 ± 0.77	26.12 ± 0.73	0.000^**^	28.02 ± 0.47	5.21	0.52	0.47	18.59	
TLA (cm^2^)	2015	31.70 ± 0.74	19.94 ± 0.61	0.000^**^	23.72 ± 0.46	5.12	0.68	0.71	21.59	89.95
	2016	34.24 ± 1.32	25.79 ± 0.76	0.000^**^	25.96 ± 0.34	3.76	0.07	−0.29	14.48	
FOLA (cm^2^)	2015	27.02 ± 0.60	18.96 ± 0.82	0.000^**^	20.17 ± 0.43	4.78	0.57	0.02	23.70	91.11
	2016	32.12 ± 1.00	21.24 ± 0.66	0.000^**^	23.12 ± 0.39	4.26	−0.10	−0.16	18.43	

^**^Significant at 0.01 level. ST: Significant; CV: Coefficient of variation. FLL, flag leaf length; SLL, second leaf length; TLL, third leaf length; FOLL, fourth leaf length; FLA, flag leaf area; SLA, second leaf area; TLA, third leaf area; FOLA, fourth leaf area.

**Table 2:** Variance analysis of top four leaves length and area traits of 122 barley DH lines, sum of squares was shown.

Source	FLL	SLL	TLL	FOLL	FLA	SLA	TLA	FOLA
Genotype	1401.620^**^	1289.296^**^	1023.262^**^	1164.586^**^	4840.289^**^	5453.042^**^	4042.199^**^	4210.667^**^
Year	321.541^*^	115.810	88.200^**^	222.114	1407.409^**^	705.840	307.038^**^	531.148^*^
Genotype × Year	261.954^**^	240.455^*^	236.675^**^	215.886^*^	902.905^**^	972.297	841.754^**^	745.826^**^

^*^Significant at 0.05 level, ^**^Significant at 0.01 level. FLL, flag leaf length; SLL, second leaf length; TLL, third leaf length; FOLL, fourth leaf length; FLA, flag leaf area; SLA, second leaf area; TLA, third leaf area; FOLA, fourth leaf area.

### Correlation analysis

The Pearson correlation coefficients (r) between topmost four leaves traits, plant height and yield-related traits were calculated based on the mean value of each year (Table 3). The top four leaf traits showed significant positive correlation with MSL, PH and TGW (p < 0.01). GP was significantly and negatively correlated with FLL (r = −0.214, P < 0.05 in 2015, r = −0.345, p < 0.01 in 2016) and FLA (p < 0.01). Significant negative correlations were also detected between SLP and FLL (p < 0.01), SLL (r = −0.216, p < 0.05 in 2015, r = −0.249, p < 0.01 in 2016), FLA (P < 0.01), SLA (r = −0.218, P < 0.05 in 2015, r = −0.235, P < 0.01) and TLA (r = −0.189, P < 0.05 in 2015). GWS was significantly positively correlated with TLL, FOLL, SLA, TLA and FOLA in 2015, and all leaf traits in 2016 (P < 0.01). Significant positive correlations between GWP and top four leaves traits except FLL, FLA, TLA and FOLA in 2016 were observed (Table 3).

**Table 3:** Correlation analysis between top four leaves traits and yield-related traits based on data from each year.

	MSL	GP	SLP	GWS	GWP	PH	TGW	FLL	SLL	TLL	FOLL	FLA	SLA	TLA	FOLA
MSL		0.048	0.042	0.359^**^	0.378^**^	0.733^**^	0.356^**^	0.278^**^	0.368^**^	0.440^**^	0.503^**^	0.246^**^	0.350^**^	0.467^**^	0.522^**^
GP	0.139		0.993^**^	0.421^**^	0.590^**^	0.195^*^	−0.725^**^	−0.214^*^	−0.139	−0.077	−0.046	−0.210^**^	−0.169	−0.140	−0.128
SLP	0.0948	0.920^**^		0.282^**^	0.442^**^	0.1015	−0.714^**^	−0.286^**^	−0.216^*^	−0.1598	−0.1117	−0.274^**^	−0.218^*^	−0.189^*^	−0.170
GWS	0.387^**^	0.328^**^	0.1405		0.734^**^	0.611^**^	0.0598	0.0507	0.1548	0.271^**^	0.332^**^	0.10753	0.199^*^	0.291^**^	0.339^**^
GWP	0.458^**^	0.676^**^	0.512^**^	0.646^**^		0.551^**^	0.1674	0.192^*^	0.250^**^	0.334^**^	0.317^**^	0.199^*^	0.245^**^	0.314^**^	0.304^**^
PH	0.761^**^	0.275^**^	0.1655	0.639^**^	0.605^**^		0.291^**^	0.307^**^	0.401^**^	0.512^**^	0.590^**^	0.316^**^	0.361^**^	0.463^**^	0.531^**^
TGW	0.353^**^	−0.584^**^	−0.658^**^	0.1643	0.1439	0.301^**^		0.493^**^	0.449^**^	0.437^**^	0.364^**^	0.469^**^	0.461^**^	0.486^**^	0.456^**^
FLL	0.288^**^	−0.345^**^	−0.445^**^	0.257^**^	0.0758	0.347^**^	0.583^**^		0.890^**^	0.748^**^	0.631^**^		0.864^**^	0.737^**^	0.646^**^
SLL	0.383^**^	−0.151	−0.249^**^	0.342^**^	0.185^*^	0.505^**^	0.448^**^	0.821^**^		0.926^**^	0.802^**^	0.812^**^		0.857^**^	0.770^**^
TLL	0.287^**^	−0.044	−0.1395	0.335^**^	0.185^*^	0.471^**^	0.293^**^	0.579^**^	0.830^**^		0.920^**^	0.697^**^	0.850^**^		0.876^**^
FOLL	0.419^**^	−0.003	−0.095	0.418^**^	0.284^**^	0.623^**^	0.351^**^	0.594^**^	0.795^**^	0.905^**^		0.621^**^	0.754^**^	0.855^**^	
FLA	0.219^**^	−0.353^**^	−0.445^**^	0.295^**^	0.0905	0.306^**^	0.587^**^		0.750^**^	0.508^**^	0.536^**^		0.887^**^	0.767^**^	0.689^**^
SLA	0.348^**^	−0.172	−0.235^**^	0.389^**^	0.182^*^	0.439^**^	0.451^**^	0.802^**^		0.679^**^	0.704^**^	0.837^**^		0.931^**^	0.840^**^
TLA	0.288^**^	−0.138	−0.1668	0.337^**^	0.1438	0.350^**^	0.363^**^	0.644^**^	0.780^**^		0.783^**^	0.679^**^	0.879^**^		0.953^**^
FOLA	0.337^**^	−0.151	−0.167	0.292^**^	0.15	0.380^**^	0.406^**^	0.615^**^	0.671^**^	0.602^**^		0.650^**^	0.815^**^	0.902^**^	

^*, **^Significant at 0.05, 0.01 level, respectively. Values above the diagonal are correlation coefficients in 2016; values below the diagonal are correlation coefficients in 2015. MSL, main spike length; GP, grain number per plant; SLP, spikelet number per plant; GWS, grain weight per spike; GWP, grain weight per plant; TGW, thousand grain weight; PH, Plant height; FLL, flag leaf length; SLL, second leaf length; TLL, third leaf length; FOLL, fourth leaf length; FLA, flag leaf area; SLA, second leaf area; TLA, third leaf area; FOLA, fourth leaf area.

### QTL analysis

In total, 57 QTLs for top four leaves length and area were identified on chromosome 2H (22 QTLs), 3H (8 QTLs), 4H (12 QTLs) and 7H (15 QTLs). Thirty-one and 26 QTLs were detected in 2015 and 2016, respectively. The individual QTL accounted for 5.17–37.11% and 5.28–35.64% phenotypic variation in 2015 and 2016, respectively (Supplementary Table S1). Among the 57 QTLs, 26 QTLs (46%) were detected in the both years.

### Flag-leaf length

A total of 29 QTLs for the leaf length traits were detected. All positive alleles were contributed by Huadamai6. Six QTLs for FLL trait over two years were identified on 2H (5 QTLs) and 7H (1 QTL) with phenotypic variations ranging from 5.30% to 37.11% (Supplementary Table S1). However, four QTLs, *qFLL2-4*, *qFLL2-5*, *qFLL2-8* and *qFLL7-2* were detected in one year and accounted for 5.30%, 12.39%, 6.62% and 9.02% of phenotypic variation, respectively. Only one common QTL *qFLL2-2* was identified on chromosome 2H across two years and explained 37.11% and 30.71% of phenotypic variations in 2015 and 2016, respectively. The major QTL *qFLL2-2* was near to the SNP marker 2HL_25536047.

### Second-leaf length

Nine QTLs for SLL trait were identified on 2H (3 QTLs), 3H (2 QTLs), 4H (1 QTL) and 7H (3 QTLs) with individual QTL accounting for 5.39–34.64% of phenotypic variations (Supplementary Table S1). QTLs *qSLL2-2, qSLL3-1* and *qSLL7-1* were identified in both years. *qSLL2-2* accounted for 15.22% (2015) and 34.64% (2016) of phenotypic variation, *qSLL3-1* explained 6.32% of phenotypic variation in 2015, and 11.51% in 2016, and *qSLL7-1* accounted for 5.49% in 2015 and 8.79% in 2016. The major QTL *qSLL2-2* was adjacent to the SNP marker 2HL_25536047. The rest of QTLs were identified in one year and accounted for 5.39–10.23% of phenotypic variations.

### Third-leaf length

For TLL, seven QTLs were detected on 2H (2 QTLs), 3H (2 QTLs), 4H (2 QTLs) and 7H (1 QTL) with individual QTL accounting for 5.50–28.25% phenotypic variations (Supplementary Table S1). The QTLs *qTLL2-2*, *qTLL3-1* and *qTLL4-2* were identified in the both years. The *qTLL2-2* explained 27.43% of phenotypic variation in 2015, and 19.74% in 2016. The major QTL *qTLL2-2* was adjacent to the SNP marker 2_451183747. *qTLL3-1* accounted for 12.04% (year 2015) and 28.25% (year 2016) of phenotypic variation. *qTLL4-2* accounted for 6.12% of phenotypic variation in 2015, and 5.50% in 2016. *qTLL7-1* accounted for 6.62% of phenotypic variation in 2015.

### Fourth-leaf length

For FOLL, seven QTLs were identified on 2H (2 QTLs), 3H (2 QTLs), 4H (1 QTL) and 7H (2 QTLs), contributing 5.28–31.26% to phenotypic variation (Supplementary Table S1). Only *qFOLL3-1* was identified in both years, and explained 16.22% (year 2015) and 31.26% (year 2016) of phenotypic variation. The major QTL *qFOLL3-1* was on the verge of the SNP marker 3_511749149. The rest of QTLs were only identified in one year and explained 5.28–21.49% of phenotypic variations.

### Flag-leaf area

In total, 28 QTLs for leaf area traits were identified. Ten QTLs for FLA trait across two years were detected with phenotypic variations explained by each QTL varying from 5.17% to 23.46% (Supplementary Table S1). These QTLs were mapped on 2H (3 QTLs), 3H (2 QTLs), 4H (3 QTLs) and 7H (2 QTLs). Six positive alleles on 2H and 7H were contributed by Huadamai6, and the positive alleles of *qFLA3-1, qFLA4-2, qFLA3-2* and *qFLA4-2* on chromosome 3H and 4H were from Huaai11. One QTL *qFLA2-2* on 2H was detected across two years and contributed 10.31% (2015) and 23.46% (2016) of phenotypic variation. *qFLA2-2* was a stable QTL nearby the SNP marker 2HL_25536047. Four QTLs, *qFLA3-1*, *qFLA4-2*, *qFLA4-3* and *qFLA7-1*, were identified in 2015 and individually explained 5.17%, 9%, 6.69% and 5.83% phenotypic variation, respectively. Four QTLs, *qFLA2-4*, *qFLA3-2*, *qFLA4-2* and *qFLA7-2*, were detected in 2016, and individually accounted for 16.83%, 6.58%, 5.46% and 6.23% phenotypic variation, respectively.

### Second-leaf area

Six QTLs for SLA trait were identified on 2H (3 QTLs), 4H (1 QTL) and 7H (2 QTLs) with individual QTL explaining 6.99–35.64% of phenotypic variations, and all positive alleles were contributed by Huadamai6 (Supplementary Table S1). The QTL *qSLA7-1* was identified in both years, and explained 6.99% of phenotypic variation in 2015 and 11.85% in 2016. *qSLA2-1*, *qSLA2-2*, *qSLA2-3* and *qSLA4-2* accounted for 19.48%, 29.12%, 35.64% and 9.27% of phenotypic variation, respectively.

### Third-leaf area

Six QTLs for TLA trait were detected on 2H (2 QTLs), 4H (2 QTLs) and 7H (2 QTLs) with phenotypic variation varying from 6.23% to 31.24% and all positive alleles originated from Huadamai6 (Supplementary Table S1). Only *qTLA4-2* was identified in both years and explained 7.05% of phenotypic variation in 2015 and 12.98% in 2016. The remaining QTLs were detected in one year and accounted for 6.23–31.24% of phenotypic variations.

### Fourth-leaf area

Six QTLs for FOLA trait were identified on 2H (2 QTLs), 4H (2 QTLs) and 7H (2 QTLs) with phenotypic variation ranging from 7.64% to 35.24%, all carried positive alleles from Huadamai6 (Supplementary Table S1). The QTL *qFOLA4-1* was identified in both years and explained 7.64% of phenotypic variation in 2015 and 14.09% in 2016. The rest of QTLs were found in one year, and *qFOLA2-3* accounted for the highest phenotypic variation up to 35.24% in 2016.

### QTL clusters for leaf length and leaf area traits

**Figure 1:**
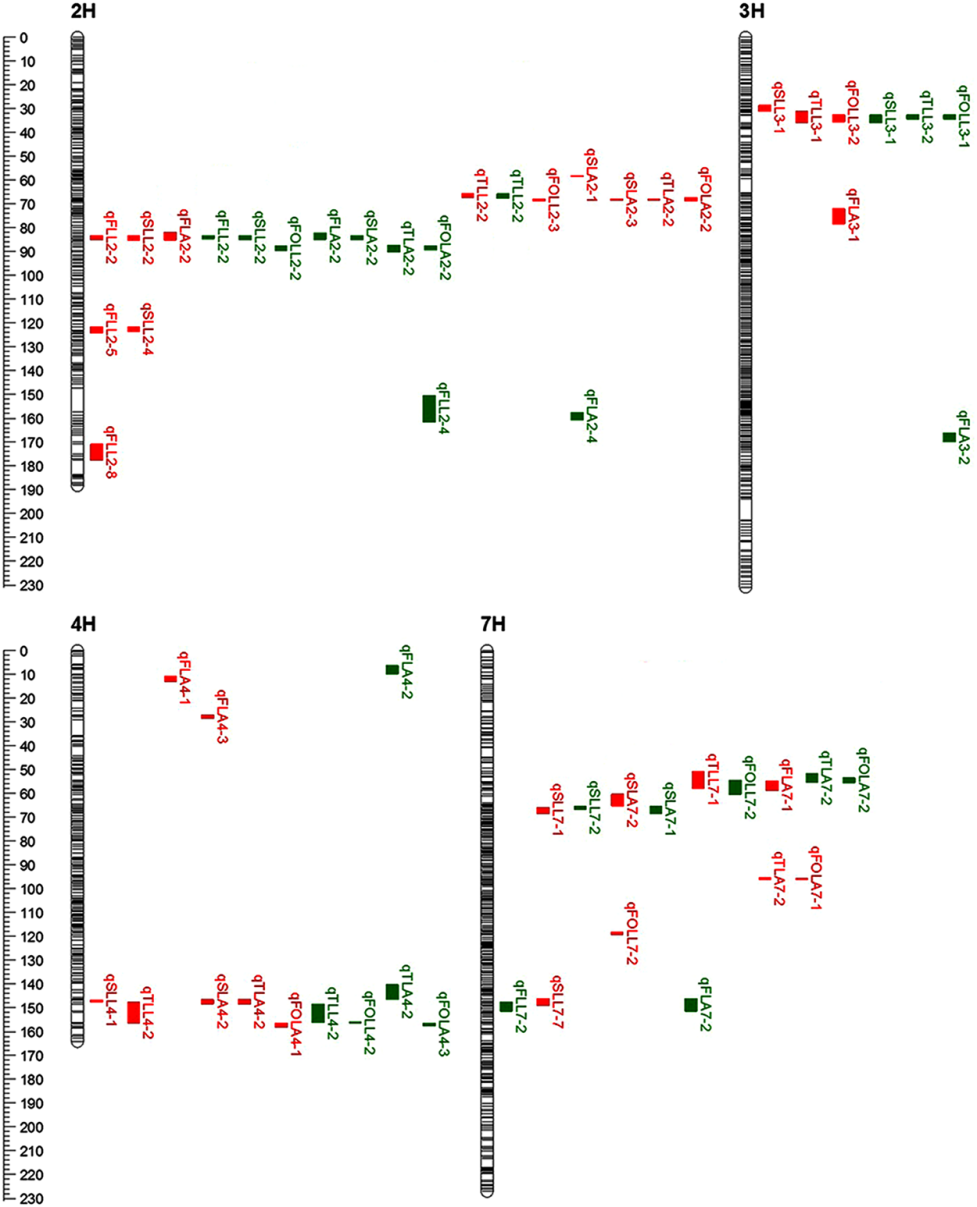
Chromosomes location of reliable QTL associated with top four leaves length and area traits. Genetic distance scale in centiMorgan (cM) was placed at left margin. The horizontal bars in the genetic map represented the position of the markers. Location of QTL was indicated for 2015 (red) and 2016 (green). FLL, flag leaf length; SLL, second leaf length; TLL, third leaf length; FOLL, fourth leaf length; FLA, flag leaf area; SLA, second leaf area; TLA, third leaf area; FOLA, fourth leaf area.

In total, 57 QTLs were detected in this population, five QTL clusters associated with top four leaf length and area traits were found on chromosome 2H (two clusters), 3H (one cluster), 4H (one cluster) and 7H (one cluster) (Fig. 1, Table 4). These QTLs increased top four leaf length and area were contributed by Huadamai6 alleles in these QTL cluster regions. Six QTLs were co-localized in C1 on chromosome 2H underlying TLL, FOLL, SLA, TLA and FOLA. The major QTL *qFLL2-2* in C2 was identified in two years and co-localized with *qSLL2-2* and *qFLA2-2*, the positive alleles from Huadamai6 increased the FLL, SLL and FLA. A major QTL for FOLL (*qFOLL3-1*) in C3 clustered with two stable QTL *qSLL3-1* and *qTLL3-1*, and the favorite alleles from Huadamai6 increased these traits simultaneously. The C4 on chromosome 4H contained stable QTLs (*qTLL4-2*, *qTLA4-2* and *qFOLA4-1*) controlling TLL, TLA and FOLA. Nine QTLs were co-localized in C5 on chromosome 7H underlying FLA, SLL, SLA, TLL, FOLL, TLA and FOLA. This QTL cluster contained two stable QTLs (*qSLL7-1* and *qSLA7-2*) controlling SLL, SLA.

**Table 4:** The QTL clusters simultaneously affecting several traits in this study.

Clusters	Chromosomes	Intervals	Position (cM)	No. of QTLs	Traits (number of years)
**C1**	2H	2HL_49027260 - 2_426554428	64.59–69.02	6	TLL(2), FOLL(1), SLA(1),
					TLA(1), FOLA(1)
**C2**	2H	M_256210_824 - 2HL_23335246	80.87–90.23	10	FLL(2), SLL(2), FOLL(1), FLA(2),
					SLA(1), TLA(1), FOLA(1)
**C3**	3H	Bmag13 - 3_504106156	28.48–35.91	6	SLL(2), TLL(2), FOLL(2)
**C4**	4H	4_42378533 - 4_8073993	139.62–158.50	9	SLL(1), TLL(2), FOLL(1), SLA(1)
					TLA(2), FOLA(2)
**C5**	7H	7_542104217 - 7HL_3360534	47.10–69.18	9	TLL(1), FOLL(1), FLA(1)
					TLA(1), FOLA(1)

FLL, flag leaf length; SLL, second leaf length; TLL, third leaf length; FOLL, fourth leaf length; FLA, flag leaf area; SLA, second leaf area; TLA, third leaf area; FOLA, fourth leaf area.

## Discussion

Leaf is the main organ of photosynthesis, and the top two leaves produce over 80% of the net photosynthesis product during grain filling, particularly the flag leaf, which assimilates most of the light energy and converts it to 41–43% of the carbohydrates for kernel filling^3,30^. Thus, leaf morphological traits, such as length, width and area, are considered as important components in determining grain yield potential. Liu *et al*.^18^ have reported the correlation and QTL of physiological and morphological traits of flag leaf in barley. However, QTL analysis related to the uppermost four leaf length and area traits has not been reported.

In our study, we detected 57 QTLs for eight leaf morphological traits: FLL, SLL, TLL, FOLL, FLA, SLA, TLA and FOLA. Six QTLs for FLL in two years were located on chromosome 2H and 7H (Fig. 1, Supplementary Table S1). A major QTL *qFLL2-2* that was stable and not influenced by environment was close to the SNP marker 2HL_25536047 on the chromosome 2H long arm. QTL for flag-leaf length was previously reported on chromosome 2H, 3H, 5H and 7H^14–18^. The QTL *qFLL2-2* was different from those QTL reported by Elberse *et al*.^14^ on 2HS, and is likely a new QTL. The QTL *qFLL2-4* on 2H and nearby the SNP marker 2HL_13648618 was associated with row number (*Vrs1*). The QTL *qFLL7-2* on 7H is different from the QTL on 7HS reported by Xue *et al*.^17^.

As expected, three coincident genetic regions were detected between FLA and FLL. For example, *qFLA2-2*, *qFLA2-4* and *qFLA7-2* were located at the same regions as *qFLL2-2*, *qFLL2-5* and *qFLL7-2*. In this study, we detected 10 QTLs associated with FLA on 2H, 3H, 4H and 7H over two years (Fig. 1, Supplementary Table S1). A stable QTL *qFLA2-2* was detected on 2H. The QTL *qFLA2-2* was close to the SNP marker 2HL_25536047 and SSR marker GBM1218. Li *et al*.^15^ detected *Qla2.1* for FLA close to the marker HVHOTR1 on 2H in a BC_3_F_2_ population. The genetic-linkage map of Varshney *et al*.^31^ illustrated the marker HVHOTR1 was near to the marker GBM1218, indicating that *qFLA2-2* was likely the same locus to the QTL *Qla2.1*.

QTL for leaf morphological traits from the second to the fourth leaf of barley has not been studied in detail. In our study, 41 QTLs associated with SLL, TLL, FOLL, SLA, TLA and FOLA were detected over two years with phenotypic variation ranging from 5.28% to 35.64%. The six traits were controlled by genetic regions on 2H, 3H, 4H and 7H (Fig. 1, Supplementary Table S1). Wherein, ten QTLs were detected in both years and included three major QTLs. These major QTLs can be used in MAS to improve the leaf morphological traits from the second leaf to the fourth leaf.

In the present study, we detected 57 QTLs associated with top four leaf length and area traits, including five QTL clusters on 2H, 3H, 4H and 7H. Our previously reported QTL for FLL and FLA in C2 on chromosome 2H also influenced those traits in other populations^15,32^. The most important QTL cluster region at the M_256210_824 - 2HL_23335246 was close to the marker GMS3 on 2H, associated with QTLs for FLL, SLL, FLA and SLA (Fig. 1, Table 4, Supplementary Table S1). QTLs affecting the chlorophyll, thousand grain weight and plant height were previously located on 2H close to the SSR marker GMS3 inferred from GrainGenes (http://wheat.pw.usda.gov/GG3/)^33–36^, which was in the same region detected here. This region also has QTL associated with grain number per spike and net photosynthetic rate, stomatal conductance and chlorophyll content of the flag leaf physiological traits previously detected in the same population^18,22,37^, indicating this QTL cluster region not only affected the morphology of leaf, but also had positive effect on the physiological traits of flag leaf.

The second noticeable clustered QTL region was Bmag13 - 3_504106156 on 3H contained *qSLL3-1*, *qTLL3-1* and *qFOLL3-1* underlying SLL, TLL and FOLL (Fig. 1, Table 4). Ren *et al*.^37^ detected QTL for heading date on 3H, which was linked with the marker Bmag13 in the same population, and near to this QTL cluster region. The QTLs underlying rachis internode length, plant height, grain yield, number of tillers and days until heading were near to the marker Bmag13 in this clustered QTL region^35,38,39^.

The third QTL cluster was in the region 4_42378533 - 4_8073993 on 4H determined SLL, TLL, FOLL, SLA, TLA and FOLA (Fig. 1, Table 4). According to GrainGenes (http://wheat.pw.usda.gov/GG3/), this region affecting ears per m^2^, heading date, thousand kernel weight, plant height and yield were located on 4H, and near to the SSR marker HVM40 in this clustered QTL region^33,35,39^. The fourth QTL cluster in the region 7_542104217 - 7HL_3360534 was close to the SSR marker GBM1102 on 7H, which was associated with FLA, SLL, SLA, TLL, FOLL, TLA and FOLA (Fig. 1, Table 4). Search against GrainGenes (http://wheat.pw.usda.gov/GG3/) found that QTLs affecting plant height, heading date and single plant yield were located on 7H adjacent to the marker GBM1102, which was similar to this region detected here^39,40^. Considerable to note was the above-mentioned four QTL cluster regions, each containing QTL-controlling plant height and heading date. Previous studies reported that plant-height and heading-date traits had significant effect on grain yield^41–43^. The co-localization of these QTLs likely resulted from closely linked QTL or pleiotropic QTL.

The leaf is the main organ of photosynthesis, in which the top three leaves, especially the flag leaf, absorb most of the plant’s light energy and is the main source of carbohydrate production^2–4^. In this study, the top four leaf traits were significantly positively correlated with yield-related traits, such as MSL and TGW, with the highest correlation coefficient between flag leaf and TGW. Significant positive correlation was also detected between flag leaf and GWS and GWP (Table 3). Previous studies have also reported the importance of flag leaf traits such as leaf length, leaf angle and leaf area on yield^6,44–46^. We detected co-localized QTL between the top four leaf traits and yield-related traits, such as MSL, SLP, GWP and TGW, on 2H, 4H and 7H^22^. Therefore, QTLs for different leaf traits co-localized with yield-related traits can be effectively utilized in MAS. In addition, significant positive correlations between the top four leaves and PH, and QTL for FLL (q*FLL7-2*) near the dwarf gene *btwd1* on chromosome 7H^23^ suggested that leaf size might be affected by plant height.

A major QTL was detected for FLL, SLL, FLA and SLA on 2HL. Blast SNP-tag sequence searched against http://floresta.eead.csic.es/barleymap using the Barleymap program^47^ to anchor the QTL marker (2HL_25536047) in the Barke × Morex POPSEQ population^48^. The QTL annotation by Barleymap program found three potentially relevant functional genes, FHY3/FAR1, gibberellin-regulated gene and SAUR gene. Tang *et al*.^49^ reported FHY3 (FAR-RED ELONGATED HYPOCOTYL3) and FAR1 (FAR-RED IMPAIRED RESPONSE1) were transcriptional factors derived from ancient transposases in evolutionary process. They were involved in chlorophyll biosynthesis via the activation of HEMB1 gene expression in *Arabidopsis thaliana*, and may have a wide range of functions in plant growth and development. Aubert *et al*.^50^ described that gibberellin-regulated, gene-encoded and gibberellin-regulated family proteins have some role in plant development. Gil and Green^51^ found that SAUR gene expressed in growing hypocotyls or other extended tissues had a certain function in regulating cell elongation. The above-mentioned genes might be considered as candidates in determining these traits. Of course, we cannot rule out the role of other genes in this QTL region. At present, the function of these genes on barley is unknown and will be characterized in future studies.

In this research, a total of 57 QTLs associated with top four leaf length and area traits were identified with individual QTL explaining 5.17% and 37.11% of the phenotypic variation. Five clustered QTL regions were detected on chromosome 2H, 3H, 4H and 7H. Ten QTLs were co-localized in the C2 cluster, such as *qFLL2-2*, *qSLL2-2*, *qFLA2-2* and *qSLA2-3*. Two major QTLs *qFLL2-2*, *qSLL2-2* and one stable *qFLA2-2* explained high phenotypic variation in this QTL cluster. Two stable QTLs *qSLL3-1*, *qTLL3-1* and one major *qFOLL3-1* on 3H detected in two years were associated with SLL, TLL and FOLL and co-localized in the C3. The C4 on 4H contained stable QTLs (*qTLL4-2*, *qTLA4-2* and *qFOLA4-1*) controlling TLL, TLA and FOLA. Five QTLs were co-localized in the C5 on 7H associated with TLL, FOLL, FLA, TLA and FOLA. These QTL clusters could be used as target regions for improving leaf morphology of barley.

## Supplementary information

Supplementary Information
