# Supplementary material for: Identification of QTL underlying the leaf length and area of different leaves in barley
Source: Sci Rep. 2019 Mar 14;9:4431. doi: 10.1038/s41598-019-40703-6 (PMC6418291; doi:10.1038/s41598-019-40703-6)
Supplement: Supplementary file 1 — Supplementary Information [file 41598_2019_40703_MOESM1_ESM.pdf]

Supplementary Information

Identification of QTL underlying the leaf length and area of different leaves in barley

Binbin Du, Lipan Liu, Qifei Wang, Genlou Sun, Xifeng Ren, Chengdao Li, Dongfa Sun*

*Corresponding author

Dongfa Sun: sundongfa1@mail.hzau.edu.cn

Supplementary Figure S1. Frequency distributions of top four leaves length and area traits in 122 doubled haploid (DH) lines. FLL, flag leaf length; SLL, second leaf length; TLL, third leaf length; FOLL, fourth leaf length; FLA, flag leaf area; SLA, second leaf area; TLA, third leaf area; FOLA, fourth leaf area.

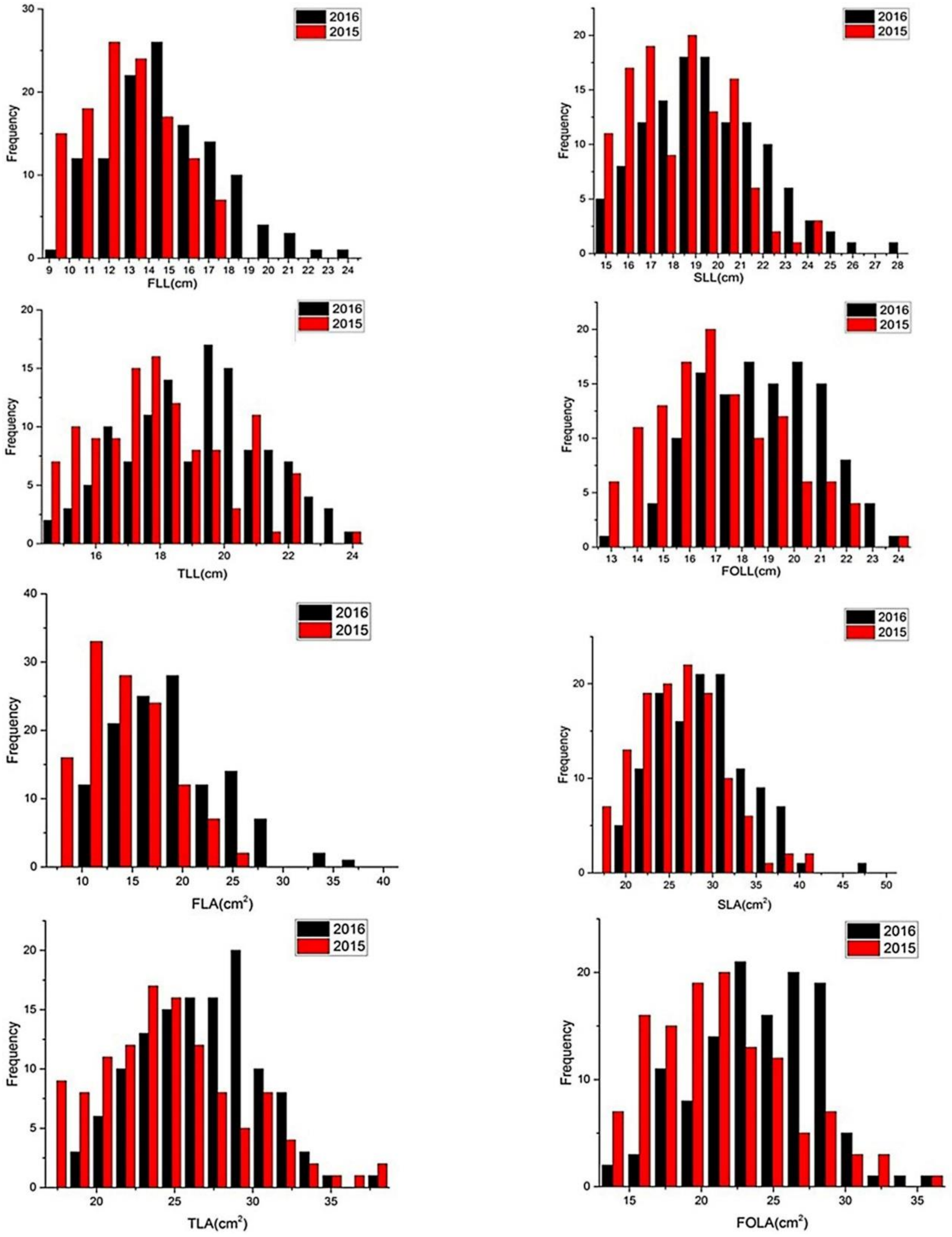

Supplementary Table S1. Credible QTLs, their locations and effects for top four leaves length and area traits. [a]2-LOD confidence interval which was determined by finding the region on both sides of a QTL peak that corresponds to a decrease of 2 LOD score.[b]A LOD threshold of 3.0 was used for declaration of QTL, based on 1000 permutations at a significance level of 0.01.[c]Phenotypic variance explained by QTL.[d]± Additive effect. Positive values indicate a positive effect of Huadamai6 alleles, whereas negative values indicate the contribution of the Huaai11 allele.FLL, flag leaf length; SLL, second leaf length; TLL, third leaf length; FOLL, fourth leaf length; FLA, flag leaf area; SLA, second leaf area; TLA, third leaf area; FOLA, fourth leaf area.

**Trait**	**QTL**	**Chr.**	**Nearest marker**	**Position (cM)**	**Interval[a]**	**LOD[b]**	**PVE(%)[c]**	**Add[d]**	**Year**
**FLL**	qFLL2-2	2	2HL_25536047	84.5	83.2 – 85.0	16.41-18.94	30.71-37.11	+	2015,2016
	qFLL2-4	2	2HL_13648618	123.5	121.6-124.3	3.46	5.3	+	2015
	qFLL2-5	2	2HL_28631837	159.81	150.5-161.5	9.33	12.39	+	2016
	qFLL2-8	2	2_588885691	175.91	170.9-177.7	4.22	6.62	+	2015
	qFLL7-2	7	7HL_6335336	150.11	147.6-151.5	7.15	9.02	+	2016
**SLL**	qSLL2-2	2	2HL_25536047	84.51	83.2-85.2	9.05-19.12	15.22-34.64	+	2015,2016
	qSLL2-4	2	2HL_13648618	123.51	121.6-123.7	3.56	5.39	+	2015
	qSLL3-1	3	3_511749149	33.71	28.6-35.9	4.15-8.62	6.32-11.51	+	2015,2016
	qSLL4-1	4	4HS_38761945	146.71	146.0-147.7	6.42	10.23	+	2015
	qSLL7-1	7	7HL_4313756	66.21	65.2-68.5	4.2-6.85	5.49-8.79	+	2015,2016
	qSLL7-7	7	5HL_40328673	146.91	146.1-149.1	4.27	5.57	+	2015
**TLL**	qTLL2-2	2	2_451183747	66.51	65.7-67.7	14.06-17.54	19.74-27.43	+	2015,2016
	qTLL3-1	3	3_511749149	33.71	31.2-35.9	8.37-17.63	12.04-28.25	+	2015,2016
	qTLL4-2	4	4HS_9277108	156.21	147.7-156.5	4.74-4.92	5.50-6.12	+	2015,2016
	qTLL7-1	7	7HL_6657809	53.21	47.9-55.1	5.27	6.62	+	2015
**FOLL**	qFOLL2-2	2	2_406934594	68.21	67.8-68.8	11.08	18.2	+	2015
	qFOLL2-3	2	Bmag829	88.11	87.5-89.7	16.05	21.49	+	2016
	qFOLL3-1	3	3_511749149	33.71	32.6-35.5	9.54-19.72	16.22-31.26	+	2015,2016
	qFOLL4-2	4	M_120736_1101	156.31	156-156.5	7.97	8.99	+	2016
	qFOLL7-1	7	M_159744_1945	55.51	51.6-57.5	4.94	5.28	+	2016
	qFOLL7-2	7	7_268311781	119.01	118.3-119.4	7.08	10.54	+	2015
**FLA**	qFLA2-2	2	2HL_25536047	84.5	81.9-85.2	6.40-15	10.31-23.46	+	2015,2016
	qFLA2-4	2	2HL_28631837	159.81	149.8-160.8	3.98-11.57	4.78-16.83	+	2015,2016
	qFLA3-1	3	Gms116	74.01	71.9-78.4	3.42	5.17	–	2015
	qFLA3-2	3	3_334498383	168.01	166.1-169.9	5.19	6.58	–	2016
	qFLA4-1	4	4_540752045	7.01	6.3-9.8	4.32	5.46	–	2016
	qFLA4-2	4	4_528451537	11.81	10.8-12.9	5.54	9	–	2015
	qFLA7-1	7	7_598211495	55.41	51.9-56.0	3.81	5.83	+	2015
	qFLA7-2	7	7HL_36320136	149.11	146.1-151.5	5	6.23	+	2016
**SLA**	qSLA2-1	2	M_193322_318	58.11	58.1-58.6	9.65	19.48	+	2015
	qSLA2-2	2	2_406934594	68.21	67.8-68.5	16.21	29.12	+	2015

	qSLA2-3	2	2_596664618	84.01	83.2-85.0	18.47	35.64	+	2016
	qSLA4-2	4	4HS_38761945	146.71	146.5-148.4	6.44	9.27	+	2015
	qSLA7-2	7	7HL_28107982	67.01	60.3-68.5	4.92-7.14	6.99-11.85	+	2015,2016
**TLA**	qTLA2-2	2	2_406934594	68.21	67.8-68.5	17.52	28	+	2015
	qTLA2-3	2	Bmag829	88.11	87.2-90.2	15.19	31.24	+	2016
	qTLA4-2	4	4_28741355	143.71	140.3-148.4	5.62-7.54	7.05-12.98	+	2015,2016
	qTLA7-2	7	M_159744_1945	55.11	51.6-55.4	3.86	6.23	+	2016
	qTLA7-3	7	4HL_39191572	95.91	95.4-96.2	8.8	11.82	+	2015
**FOLA**	qFOLA2-2	2	2_406934594	68.21	67.3-68.8	7.82	10.31	+	2015
	qFOLA2-3	2	Bmag829	88.11	87.5-89.3	21.52	35.24	+	2016
	qFOLA4-1	4	M_120736_1101	156.71	156.5-158.1	5.74-10.43	7.64-14.09	+	2015,2016
	qFOLA7-1	7	7_598211495	55.41	53.4-55.5	7.03	8.6	+	2016
	qFOLA7-2	7	4HL_39191572	95.91	95.7-96.1	12.32	18.16	+	2015
